# Comparing pre- and postoperative etoricoxib administration versus only postoperative on third molar extraction sequelae and oral health quality of life: a prospective quasi-experimental study

**DOI:** 10.1007/s00784-024-05614-5

**Published:** 2024-03-15

**Authors:** Giusy Rita Maria La Rosa, Matteo Consoli, Roula S. Abiad, Angelo Toscano, Eugenio Pedullà

**Affiliations:** 1 Department of General Surgery and Medical-Surgical Specialties, University of Catania, Catania, Italy; 2 Endodontic Division, Faculty of Dentistry, Beirut Arab University, Beirut, Lebanon; 3 Private Practice, Acireale, Catania, Italy

**Keywords:** Third molar, Etoricoxib, Oral health quality of life, Oral surgery, Pain management, Analgesia

## Abstract

**Objectives:**

This study aimed to compare the impact of pre- and postoperative etoricoxib administration versus only postoperative on third molar extraction sequelae and oral health quality of life.

**Materials and methods:**

This prospective quasi experimental study involved 56 patients, divided into a study group receiving preemptive etoricoxib 120 mg before surgery and postoperative etoricoxib 120 mg (*n* = 28), and a control group receiving preemptive placebo before surgery and postoperative etoricoxib 120 mg (*n* = 28).

Follow-up assessments were conducted at 3- and 7-days post-surgery, recording swelling, trismus, and adverse events. Patients rated perceived pain using the visual analog scale (VAS) and completed an oral health-related quality of life (OHRQoL) questionnaire at specified intervals. Statistical analysis employed non-parametric tests (i.e., the Mann–Whitney test, Friedman test, and Wilcoxon sign test) with *P* < 0.05.

**Results:**

Significantly lower VAS scores were reported in the study group throughout the follow-up period (*P* < 0.05). Pharmacological protocol did not have a significant impact on postoperative edema and trismus (*P* > 0.05). However, double etoricoxib intake significantly improved postoperative quality of life on day 3 after surgery (*P* < 0.05).

**Conclusions:**

Pre- and postoperative etoricoxib 120 mg intake in third molar surgery reduced postoperative pain and enhanced postoperative quality of life on day 3 after surgery. Importantly, it was equally effective in managing swelling and trismus compared to exclusive postoperative intake.

**Clinical Relevance:**

Preemptive etoricoxib use may decrease patient discomfort following impacted mandibular third molar extraction.

## Introduction

Surgical extraction of third molars is one of the most frequently performed procedures in oral surgery. Similarly, like any other oro-maxillofacial surgical procedure, it represents a traumatic event capable of inducing varying degrees of inflammation in the postoperative period, associated with typical sequelae such as pain, edema, trismus, and other inflammatory complications [1]. Factors contributing to the onset of these sequelae include surgical technique, extent of osteotomy, and duration of the surgical procedure [2]. Additionally, the production of proinflammatory mediators such as prostaglandins, platelet-activating factor, and leukotrienes [3] can cause vasodilation and hyper-vascularization of the surgical site [4].

Edema, trismus, pain, and delayed healing can adversely impact the patient's quality of life (QoL) [5]. These complications may significantly lead to a deterioration in the QoL during the immediate postoperative period [6].

Various strategies can be employed to alleviate postoperative symptoms following third molar extraction, including the use of postoperative medications to inhibit the release of proinflammatory mediators [7]. Preemptive analgesia, considered capable of preventing peripheral and central sensitization [8], is also a viable option. Corticosteroids and non-steroidal anti-inflammatory drugs (NSAIDs) are the most commonly used analgesic and anti-inflammatory drugs [9, 10].

Numerous mediators contributing to inflammation are known, including histamine, serotonin, short and long peptides (bradykinin and interleukin 1), prostaglandins, and enzymes released by migratory cells and the complement system [11]. The primary mechanism of action of NSAIDs is the inhibition of the enzyme cyclooxygenase (COX), also known as prostaglandin H synthase, which exists in two isoforms: COX-1 (housekeeping enzyme) and COX-2, expressed by cells involved in inflammation. Both are responsible for converting arachidonic acid into thromboxanes, prostaglandins, and prostacyclins [12]. NSAIDs belonging to the COX-2 selective class only target this isoform, sparing COX-1, which is constitutively expressed in the gastrointestinal tract, thus reducing the typical side effects of non-selective NSAIDs [13, 14].

Etoricoxib is a second-generation cyclooxygenase-2 inhibitor indicated for the short-term relief of pain following dental surgery [15]. A recent meta-analysis assessed its clinical efficacy compared to traditional NSAIDs in managing postoperative pain following third molar surgery, demonstrating that at a dosage of 120 mg, it possesses superior analgesic activity compared to traditional NSAIDs, such as ibuprofen and diclofenac [16]. Furthermore, a recent double-blind randomized clinical trial reported that preoperative administration of 120 mg etoricoxib improved the postoperative course of patients, enhancing their quality of life [6].

Currently, no studies in the literature have evaluated the impact of pre- and postoperative administration of etoricoxib compared with its exclusive postoperative administration. Thus, the aim of this quasi-experimental study was to evaluate the efficacy of pre- and postoperative administration of etoricoxib 120 mg in managing postoperative sequelae of third molar surgery and its influence on patients' quality of life. The null hypotheses were that (i) there are no differences between the two treatments for managing postoperative sequelae, including pain, postoperative swelling and trismus, and (ii) there is no difference between the two treatments in increasing the oral health-related quality of life (OHRQoL).

## Materials and methods

### Study design and ethical considerations

The study was structured as a prospective quasi-experimental study. This study adhered to the 1968 Declaration of Helsinki on Medical Research and its subsequent amendments. Ethical approval was obtained from the Institutional Review Board of the University of Beirut Arab University, Beirut, Lebanon (2023-H-0133-D-R-0572). Written informed consent was obtained from each patient, who was informed about the study protocol, including the blinded clinical study design, surgical procedure, and drugs administrated. Hypothetical short- and long-term risks including surgical procedure-related injuries and possible drug-related allergic reactions or adverse events, were also referred before any procedure started. The study was conducted following TREND (Transparent Reporting of Evaluations with Nonrandomized Designs) guidelines [17].

### Inclusion and exclusion criteria

All patients were consecutively recruited from healthy subjects, aged 18 years or older, with an indication for the surgical removal of an impacted mandibular third molar. Patient recruitment took place in February 2023 at a reference clinic in Acireale, CT, Italy. Participants were eligible if all inclusion criteria were met: (1) age 18 years or older; (2) absence of systemic diseases with patients classified as ASA I according to American Society of Anesthesiologists (ASA) classification criteria; (3) abstinence from the use of analgesic, anti-inflammatory, and/or antipyretic drugs in the previous seven days; (4) absence of patient allergy to the molecule and/or excipients of the drugs used; (5) absence of infectious foci at the surgical site and/or absence of symptoms; and (6) mandibular third molars with complete clinical-radiographic indication of disodontiasis of the included or semi-included mandibular third molar intended for extraction [10, 18, 19]. Ineligibility for study participation was verified if participants met at least one of the following exclusion criteria: (1) pregnant or breastfeeding; (2) history of gastrointestinal ulcer or peptic ulcer events; (3) allergy to aspirin or other non-steroidal anti-inflammatory drugs; (4) systemic diseases affecting the kidneys, liver, blood, or central nervous system; (5) abuse of psychostimulant substances, analgesics, steroids, and/or non-steroidal anti-inflammatory drugs; and (6) use of contraceptives [10, 18, 19]. Patients were excluded if they did not complete the study or strictly adhere to the study protocol. Additionally, patients were excluded if the surgical procedure exceeded 40 min.

### Sample size analysis and procedures

Based on the mean pain scores of a previous study [19], a minimum sample size of twenty-eight patients per group was used to conduct this clinical trial and statistically reject the null hypothesis with 95% power. For this sample calculation, the type 1 error associated with the test was 0.05. Pain was the primary variable selected for the analysis.

**Fig. 1:**
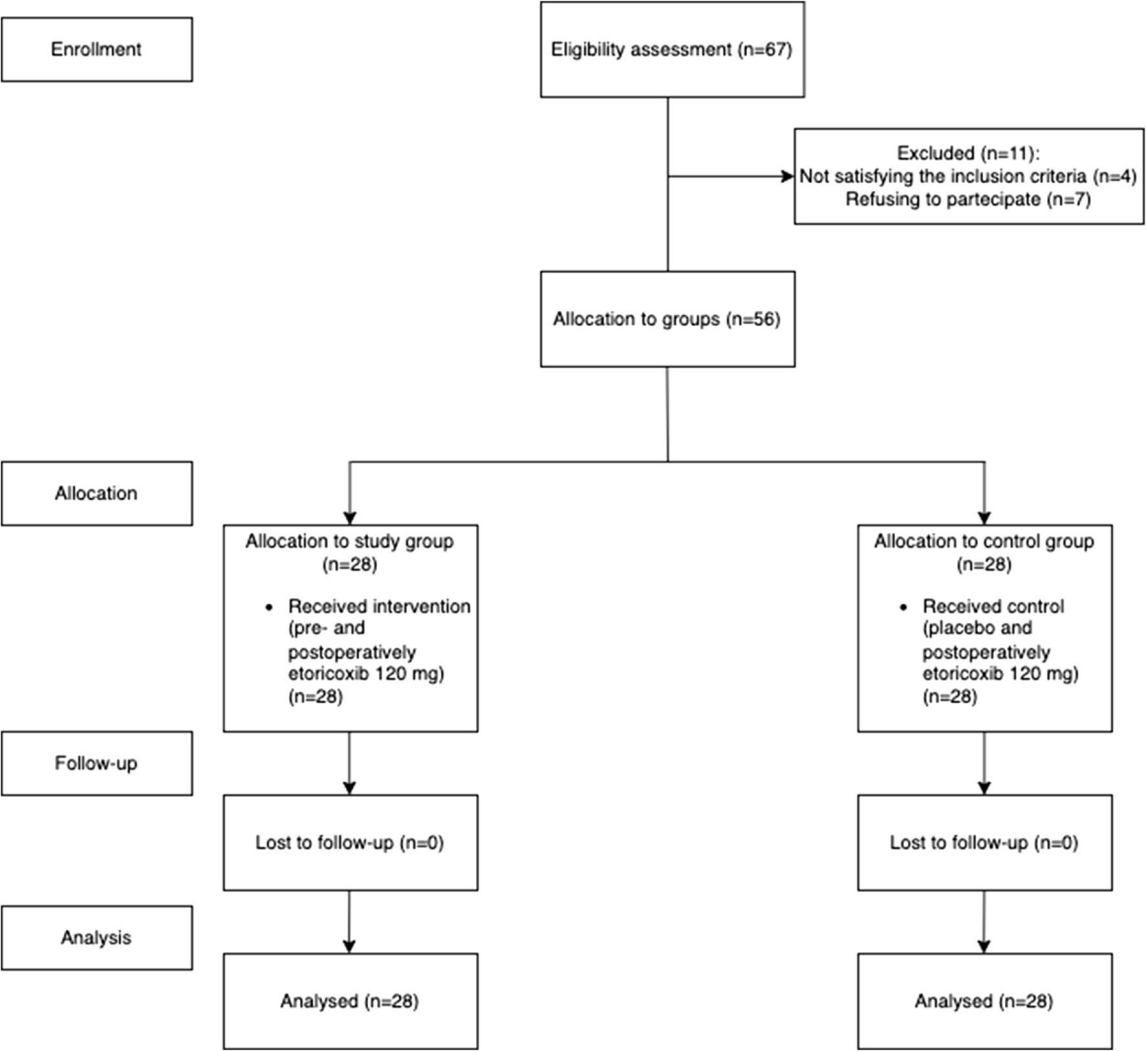
Flowchart of patients’ enrollment

During the initial phase of the research, 67 patients (35 males and 32 females) were selected from individuals referred to the reference clinic in Acireale, Italy. Following the application of the study criteria, 11 patients (6 males and 5 females) were excluded. In particular, four did not meet eligibility criteria and 7 declined participation. Consequently, the analysis comprised a total of 56 patients (29 males and 27 females) (Fig. 1).

All patients underwent an initial preoperative screening visit conducted by the same experienced physician who was blinded to the patient's group assignment. Patient data including age, sex, systemic diseases, and periodontal status were recorded prior to the procedure. Panoramic radiographs obtained before enrollment were examined to reassess the tooth position, degree of impaction, and degree of development of the teeth and roots of each third molar. Each enrolled patient was assigned to one of the 2 groups based on the timing of drug administration: the exclusively postoperative administration group (*n* = 28) received a placebo capsule (sugar pills, Olcelli farmaceutici S.r.l, Monza, Italy) preoperatively and etoricoxib 120 mg therapy twice daily for 5 days postoperatively (control group); the pre- and postoperative administration group (n = 28) received 120 mg of etoricoxib 30 min before the surgical procedure and twice daily for 5 days postoperatively (study group). Before the procedure, each patient received 1 g of amoxicillin and clavulanic acid as preoperative prophylactic therapy 1 h before the procedure (Augmentin, GlaxoSmithKline, Milan, Italy). Postoperative antibiotics, including amoxicillin and clavulanic acid, were prescribed twice a day for five days (Augmentin).

### Allocation concealment

Before initiating each treatment, an operator, not involved in subsequent study phases or data processing, managed the allocation process based on the clinical file number. Even numbers were designated to the control group, while odd numbers were allocated to the study group. Allocation concealment was maintained using a sealed opaque envelope. For the codification of the groups, the letter “A” was assigned to the placebo treatment while “B” to the study group. A clinical operator not involved in the subsequent study phases prepared the study drugs for the clinic nurses according to the predetermined coding. A dedicated nurse, not involved in the research team, gave the study drugs sealed in a similar package to the patients 30 min before the surgery [19]. Throughout follow-up sessions, the blinding protocol was sustained, with the patient, clinician, surgeon, and statistician remaining unaware of the treatment data [10].

### Surgical procedure and postoperative instructions

All procedures were performed by the same blinded clinician blinded to the assigned group to avoid potential bias related to surgeon variability. All patients underwent the same surgical extraction procedure. Local anesthesia technique involved inferior alveolar nerve block using 2% mepivacaine without adrenaline (Septodent S.r.l.; Matarò; Spain) and the subsequent local anesthesia with plexus technique using 1:100.000 articaine with adrenaline (Septodont S.r.l.). The total amount of local anesthetic used for the procedure was recorded for each patient by counting the number of dental cartridges used. All patients underwent the same full-thickness mucoperiosteal triangular flap technique with subsequent osteotomies. The bone was removed using a round bur on an angled hand piece under continuous saline irrigation. All patients underwent tooth sectioning (i.e., odontotomy) using tungsten carbide burs (MEDICALINE; Monfalcone, Italy) and removal of the third molar, followed by alveolar cavity curettage. The surgical wound was closed with a resorbable 4–0 suture (Vicryl-coated polyglactin 910; Ethicon). Immediately after the procedure, the postoperative therapy was explained to each patient in detail. Patients were instructed to follow a liquid and cold diet for the first 24 h and were also informed about the oral hygiene instructions and possible symptoms resulting from the surgical procedure. Furthermore, all possible surgical complications, such as pain, swelling, and fever, as well as the risks associated with therapy, including nausea, vomiting, or drug intolerance were explained in detail. At the end of the procedure, all patients were instructed to apply ice packs at the surgical site. Throughout the study, the surgical team assisted the patients in case of any postoperative issues such as infections, uncontrolled pain, fever, or other procedure-related complications, as needed. Any adverse reactions to drugs were recorded during each follow-up session.

### Outcomes and data collection

Immediately after the procedure, the details of each operation and total duration of the procedure were recorded. The primary outcome was the extent of pain. This allowed the patients to describe their discomfort more objectively. The intensity of the pain variable was recorded using a 10-cm visual analog scale (VAS), ranging from 0 (no pain) to 10 (maximum pain). Each patient was asked to rate the perceived pain at 2, 6, 24, 48, and 72 h, and at 5 and 7 days postoperatively. During this phase, data on any additional analgesics or other drugs taken by each participant were collected. Postoperative swelling was the second outcome investigated. For the analysis of this outcome, the preoperative and postoperative values (obtained at each follow-up session, postoperative, and at 3 and 7 days) of various facial measurements were compared, as described below: from the mandibular angle to the tragus (MA-Tr distance), from the mandibular angle to the external corner of the eye (MA-ECE distance), from the mandibular angle to the nasal edge (MA-NB distance), from the mandibular angle to the labial commissure (MA-LC distance), and from the mandibular angle to the pogonion (MA-Pg distance) [20]. For the clinical analysis of the third outcome (i.e., trismus), the maximum degree of mouth opening was determined. This measurement was performed at baseline, 30 min after the surgical procedure, and at 3 and 7 days after the surgical procedure using a calibrated sliding caliper (Vinabo S.r.l). The fourth outcome was the OHRQoL, which was evaluated by administering a questionnaire to the patient, structured with 16 questions grouped into four domains, asking them to score each question from 1 (worst discomfort condition) to 5 (best comfort condition) [21]. This allowed the patients to express their postoperative discomfort as objectively as possible. The questionnaire was administered to the patients at 24 and 48 h and at 5 and 7 days.

### Statistical analysis

Following the Kolmogorov–Smirnov test, the data were statistically examined using a non-parametric approach. The two groups were compared for baseline characteristics (i.e., gender and age) to confirm the homogeneity of groups. The Mann–Whitney test was applied for pairwise comparisons, with the significance level set at *P* ≤ 0.05. The Friedman test was used for repeated measurements over time in each group. For multiple comparisons over time, the Wilcoxon sign test was used. Statistical analyses were performed using Stata version 17 software (StatsCorp, TX, USA).

## Results

### Patient population

All enrolled patients completed the study without postoperative complications. The two groups were comparable at baseline with no significant difference regarding the age and gender (*P* > 0.05). The average age of the 56 eligible patients (29 males, 27 females) was 35.95 ± 9.89 years. Twenty-eight patients (16 males, 12 females) were assigned to the etoricoxib 120 mg group, administered in two pre- and postoperative intervals, while 28 patients (13 males, 15 females) were assigned to the etoricoxib 120 mg single postoperative interval group. Postoperative healing exhibited no negative outcomes, with no adverse events, such as infections or abscesses, during the follow-up. Additionally, no severe adverse events (e.g., nausea, vomiting, headache, tachycardia,) were reported following medication use in any follow-up session. The amount of anesthetic used and the duration of surgery were comparable between the two groups (*P* > 0.05). Osteotomy and tooth sections were performed without incidents or intraoperative complications in all enrolled patients. No alterations in sensitivity involving the inferior alveolar nerve were noted, as all patients regained normal sensitivity in the lip area after the procedure.

### Swelling

Mean swelling values were higher in the control group. Nevertheless, although clinically different swelling patterns were observed in individual extra-oral projections between the two groups, no statistical significance was found in facial measurements preoperatively, postoperatively, and at 3 and 7 days (*P* > 0.05) (Table 1).

**Table 1:** Facial distance measurements for each group at different follow-ups

Facial distances measurements (mm) Mean (SD) Median [range]			
	Study group	Control group	*p*
Baseline			
MA-Tr	57.3 (11.02) 54.25^a^ [52.1;60]	61.18 (6.38) 63.65^a^ [55.2;65.1]	0.190
MA-ECE	90.46 (14.40) 88.85^a^ [81.1;91.2]	96.68 (7.89) 95.45^a^ [93.2;98.1]	0.071
MA-NB	99.74 (14.62) 99.2^a^ [92.2;103.9]	104.08 (11.10) 102.75^a^ [95.5;111.4]	0.481
MA-LC	85.05 (16) 80.5^a^ [75.1;87.2]	83 (6.29) 81.65^a^ [78.1;87.7]	0.684
M-Pg	105.87 (18.21) 98.7^a^ [92.9;118.3]	112.19 (7.74) 109.75^abc^ [106.3;117.6]	0.217
**30 min**			
MA-Tr	63.82 (12.62) 61.8^b^ [56;70]	68.64 (6.76) 69.4^b^ [65.4;74.2]	0.191
MA-ECE	97.02 (15.61) 92.9^b^ [86.3;100.2]	102.83 (7.04) 102^b^ [100.5;110.4]	0.063
MA-NB	105.07 (16.04) 103.8^b^ [93.4;109.7]	111 (12.16) 112.35^b^ [97.3;120.6]	0.352
MA-LC	89.98 (18.69) 84.15^bc^ [80.5;92]	90.23 (7.41) 92.75^b^ [82.9;96.2]	0.305
M-Pg	110.51 (20.36) 104.35^b^ [93.6;122]	117.88 (8.71) 116.95^d^ [109.9;124.6]	0.123
**3 days**			
MA-Tr	60.51 (11.78) 58.45^a^ [53.8;66]	65.51 (6.08) 66^c^ [62.3;70.8]	0.165
MA-ECE	93.4 (14.48) 90.35^a^ [84.9;96.9]	99.43 (7.01) 99^c^ [96.1;100.6]	0.062
MA-NB	102.82 (15.48) 101.65^c^ [93.2;106.6]	107.9 (11.56) 108.15^c^ [96.7;115.2]	0.315
MA-LC	86.76 (18.12) 80.05^ac^ [76.8;86.7]	87.2 (7.17) 88.15^c^ [81.3;93.7]	0.271
M-Pg	107.51 (18.85) 101.08^a^ [92.9;117.9]	115.14 (8.44) 114.65^b^ [107.2;120.9]	0.143
**7 days**			
MA-Tr	57.82 (11.02) 55.1^a^ [53.2;59.2]	61.66 (6.60) 63.8^a^ [55.1;66.4]	0.279
MA-ECE	90.62 (13.25) 89.4^a^ [82.3;93.8]	96.92 (7.66) 95.65^a^ [94.1;98.4]	0.089
MA-NB	100.21 (15.53) 99.55^a^ [92.1;103.2]	104.5 (11.09) 102.85^a^ [95.4;112.9]	0.352
MA-LC	85.17 (15.84) 80.3^a^ [75.7;87.6]	83.55 (6.50) 83.4^a^ [78.1;88.2]	0.630
M-Pg	106 (18.18) 98.85^a^ [92.7;112.1]	112.36 (7.78) 110.02^c^ [106;117.7]	0.217

Study group (double intake; pre-and postoperative etoricoxib’s assumption); Control group (double intake; preeemptive placebo’s assumption and postoperative etoricoxib’s assumption)

ECE indicates external corner of the eye; LC, labial commissure; MA, mandibular angle; NB, nasal border; SP, soft pogonion; Tr, tragus

^*^statically significant difference *p* < 0.05

^a,b,c,d^Different superscript letters indicate statistically significance *p* < 0.05 between the different time points for the same measure

### Trismus

Mean maximum mouth opening values were lower in the control group and peaked in both groups postoperatively. However, no significant differences were found between the two groups at any time interval (*P* > 0.05). Instead, within the same group, a significant difference was noted in the maximum mouth opening size preoperatively compared to that recorded postoperatively and at 3-day for the single postoperative administration group (*P* < 0.05) (Table 2).

**Table 2:** Comparison of maximum mouth opening (trismus) for each group at different follow-ups

Maximum mouth opening (trismus) (mm) Mean (SD) Median [range]			
	Study group	Control group	*p*
Baseline	37.37 (10.46) 38.6^a^ [30.3;46.5]	33.02 (6.18) 32.45^a^ [27.4;38.2]	0.225
30 min	30.4 (12.35) 35.6^a^ [18.7;36.9]	21.67 (5.98) 22.5 ^b^ [15.9;25.6]	0.118
3 days	34.18 (9.03) 34.95^a^ [26.7;39.2]	29.07 (6.23) 29.1 ^c^ [23.2;33.6]	0.217
7 days	38.76 (7.26) 37.1^a^ [31.7;44.9]	32.89 (6.26) 33.2^a^ [27.9;37.8]	0.118

Study group (double intake; pre- and postoperative etoricoxib’s assumption); Control group (double intake; preeemptive placebo’s assumption and postoperative etoricoxib’s assumption)

^*^statically significant difference *p* < 0.05

^a,b,c^Different superscript letters indicate statistically significance *p* < 0.05 between the different time points for the same measure

### Pain

The collected data indicated that postoperative pain was lower in the study group compared to the control group throughout the postoperative week until equalizing on the seventh day. The peak of postoperative pain was observed at 6 h post-surgery in both groups. However, treatment with etoricoxib 120 mg in pre- and postoperative intervals resulted in a significant reduction in postoperative pain at 2, 6, 24, 48, and 72 h (*P* < 0.05) compared to single postoperative administration (Table 3). No significant differences were observed at 5 and 7 days (*P* > 0.05).

**Table 3:** Visual analogue scale values for each group at different follow-ups

Visual analogue scale (VAS) Mean (SD) Median [range]			
	Study group	Control group	*p*
Baseline	0 (0) 0^ab^ [0]	0 (0) 0^a^ [0]	1.000
2 h	2.65 (1.88) 3 ^abc^ [1;4]	4.9 (1.28) 4.5 ^b^ [4;5]	0.010*
6 h	3.1 (1.96) 3 ^abc^ [2;4]	5.4 (1.34) 6 ^b^ [4;6]	0.008*
24 h	2.9 (1.52) 3 ^c^ [2;4]	4.1 (0.99) 4 ^b^ [4;5]	0.044*
48 h	1.6 (1.42) 1^abc^ [1;2]	3.75 (1.81) 4 ^bc^ [2;4]	0.012*
72 h	0.4 (0.60) 0^ab^ [0;1]	2 (0.94) 2^ac^ [2;3]	0.002*
5 days	0.1 (0.31) 0^ab^ [0;0]	0.6 (0.69) 0.5^a^ [0;1]	0.119
7 days	0 (0) 0^ab^ [0;0]	0 (0) 0^a^ [0;0]	1.000

Study group (double intake; pre- and postoperative etoricoxib’s assumption); Control group (double intake; preeemptive placebo’s assumption and postoperative etoricoxib’s assumption)

^*^statically significant difference *p* < 0.05

^a,b,c^Different superscript letters indicate statistically significance *p* < 0.05 between the different time points for the same measure

### OHRQoL score

When comparing the two groups for the average scores extracted from the OHRQoL questionnaire, the pre- and postoperative administration group reported significantly higher OHRQoL values compared to the solely postoperative administration group at 3 and 7 days (*P* < 0.05), with no significant differences at 1 and 5 days (*P* > 0.05) (Table 4).

**Table 4:** Oral health related quality of life (OHRQoL) for each group at different follow-ups

OHRQoL Mean (SD) Median [range]			
	Study group	Control group	*p*
1 day	44 (4.64) 42^abc^ [41;48]	41.7 (2.71) 42^abc^ [40;42]	0.313
3 days	52.3 (4.69) 51.5 ^b^ [48;54]	43.5 (2.41) 43 ^be^ [41;45]	0.001*
5 days	52.2 (5.47) 52 ^ce^ [50;54]	50.9 (3.34) 51.5 ^ce^ [48;54]	0.781
7 days	54.3 (2.11) 55 ^de^ [53;56]	51.2 (2.61) 50.5 ^de^ [49;54]	0.013*

Study group (double intake; pre- and postoperative etoricoxib’s assumption); Control group (double intake; preeemptive placebo’s assumption and postoperative etoricoxib’s assumption)

^*^statically significant difference *p* < 0.05

^a,b,c,d,e^ Different superscript letters indicate statistically significance *p* < 0.05 between the different time points for the same measure

## Discussion

Among the various approaches to minimize surgical sequelae and ensure a better postoperative course, prescribing appropriate drug therapy, in conjunction with a congruous and as atraumatic as possible surgical procedure, is essential [22, 23]. A commonly adopted pharmacological strategy for managing postoperative complications, particularly pain following extraction of the included third molars, involves the systemic administration of NSAIDs [24]. However, several studies have reported adverse effects in patients taking NSAIDs after surgery [25–27]. Olmedo et al.'s study [28] revealed that 37.3% of patients taking non-COX-selective NSAIDs (ketorolac or ketoprofen) after third molar surgery experienced adverse events, including drowsiness (10.7%), heartburn (10.3%), and gastric injury (8%).

Building on these findings, numerous clinical trials have aimed to identify drugs with analgesic effects equivalent to NSAIDs but with fewer side effects. Studies by Shi et al. [29] and Boonriong et al. [30] suggested that the anti-inflammatory and analgesic efficacy is primarily due to inducible COX-2 inhibition, while adverse effects seem related to constitutional COX-1 inhibition. Thus, the use of selective COX-2 inhibitors (coxibs), such as celecoxib and etoricoxib, could offer advantages in dental pain management.

To date, no clinical trials have assessed systemic pre- and postoperative COX-2 selective administration to reduce postoperative complications affecting patients' quality of life. Therefore, this quasi-experimental study aimed to evaluate the impact of pre- and post- versus postoperative administration of etoricoxib alone in managing postoperative sequelae associated with mandibular third molar avulsion.

The drug dosage chosen for this trial was based on that of previous studies [16, 19, 31]. The study reported statistically significant results in the double-intake group for controlling pain, while no statistically significant differences were observed in controlling edema and trismus. Consequently, the first null hypothesis is partially rejected. Concerning OHRQoL, significant differences were observed between the two groups, leading to the rejection of the second null hypothesis.

More specifically, the data revealed a peak in postoperative edema in both study and control groups, followed by a slight decrease at the 3-day follow-up until resolution on day 7. This aligns with the edema's pathophysiology, peaking within 48 h, followed by resolution around 30 h after surgery, reaching complete resolution by the end of the first postoperative week [23, 32–34]. Mean swelling values were clinically higher in the control group than in the study group. However, despite clinically different swelling in individual extra-oral projections between the two groups, statistical significance was not evident, possibly due to the reduced sample size. Assessing the trend of individual swelling intervals in both study and control groups revealed a statistically significant difference when comparing preoperative and postoperative measures, regardless of the group. In the control group, a significant difference was found between preoperative and day 3 measurements in MA-Tr, MA-ECE, and MA-LC projections, indicating greater swelling at 3-day in patients undergoing only postoperative therapy compared to those undergoing both preoperative and postoperative etoricoxib therapy. Higher records observed on average for the control group could be attributed to the effectiveness of preemptive analgesia in reducing inflammatory mechanisms induced by the incision and trauma of surgery [19]. This aligns with previous studies demonstrating etoricoxib's efficacy in reducing arachidonic acid release and clinical swelling [35, 36]. In both groups, values tended to return to preoperative levels by day 7, suggesting the absence of late complications.

In both study and control groups, trismus peaked postoperatively, followed by a gradual improvement, resolving by day 7. The control group showed clinically lower mean maximum mouth opening than the study group, but the difference lacked statistical significance in direct comparison. However, within the control group, a significant decrease in mouth opening was observed postoperatively and at the 3-day follow-up. Etoricoxib's efficacy could be attributed to reduced arachidonic acid release, resulting in reduced inflammation and clinically reduced lockjaw [35, 36]. The lower recordings obtained on average compared with patients in the pre- and post- groups may be due to the efficacy of preventive analgesia in reducing inflammatory mechanisms induced by the incision and trauma of surgery [19]. These results align with Tiigimae-Saar et al.'s study [37] in which patients received etoricoxib 120 mg pre- and postoperatively in combination with prednisolone, showing significant improvement in swelling and reduction in trismus after third molar surgery.

Additionally, the study data indicated that treatment with etoricoxib at preoperative and postoperative administration resulted in a significant reduction in postoperative pain at 2, 6, 24, 48, and 72 h after surgery compared with postoperative administration alone. These findings align with the typical evolution of pain in third molar avulsion, appearing 2–3 h after surgery, peaking in the first 24 postoperative hours, and gradually decreasing until disappearing within the postoperative week [23, 32–34, 38]. The data also demonstrated lower postoperative pain in the study group than in the control group throughout the postoperative week, equaling assessment on day 7. A statistically significant difference was found within the pre- and post groups in postoperative pain values recorded at 24 h after surgery, characterized by peak pain, compared with those recorded at 72 h, 5 days, and 7 days, where pain values comparable to zero were recorded. The preventive administration of etoricoxib 120 mg may be effective in managing postoperative pain, consistent with Albuquerque et al.'s study [39], which evaluated the preventive analgesic efficacy of ibuprofen compared with etoricoxib, and Xie et al.'s study [6], reporting the efficacy of preoperative oral etoricoxib administration (i.e., 120 mg) in providing postoperative analgesia and improving the patient's quality of life after surgery. These results are in agreement with other studies demonstrating the efficacy of etoricoxib 120 mg before surgery in reducing the onset of postoperative pain in patients undergoing certain oral procedures or third molar surgery [19, 40, 41].

Based on the current results, a significant improvement in patients' quality of life was observed at 3 days in the study group compared to the control group. These results agree with studies by Xie et al. [6] and Bhuvan Chandra et al. [42], reporting that proper preoperative and postoperative analgesic therapy can reduce the negative impact of surgery on patients' quality of life. The mean scores were, on average, higher in the study group than in the control group on all follow-ups. However, the comparison results significant only at 3 and 7 days. These results should be interpreted with caution due to the type of test used, which implies a certain degree of subjectivity.

Further studies are desirable to confirm these preliminary results. Etoricoxib was found to be a safe and easy-to-use drug for the management of post-surgical discomfort after third molar surgery. In addition to its reported efficacy on postoperative sequelae, etoricoxib has fewer side effects, such as heartburn, and gastrointestinal effects, such as upper and lower tract lesions, compared to other NSAIDs [43]. It is also effective for pain prevention in patients after periodontal surgery [44].

As this is the first study evaluating the efficiency of a protocol based on 120 mg etoricoxib with pre- and postoperative administration, a direct comparison with other studies is not possible. This study has some limitations, including a lack of randomization and a limited sample size. The data are restricted to the tested dose and protocol applied; thus, any generalizability of the results is not allowed. Larger randomized clinical trials are necessary to confirm these preliminary results. The findings are clinically relevant because they suggest that preemptive etoricoxib use may decrease patient discomfort following impacted mandibular third molar extraction.

The findings are clinically relevant because they suggest that preemptive etoricoxib use may decrease patient discomfort following impacted mandibular third molar extraction, offering a potential improvement in post-operative care and overall patient experience.

## Conclusions

Within the limitations of this quasi-experimental study, preoperative and postoperative etoricoxib-based administration at a dosage of 120 mg significantly improved the follow-up of variables associated with postoperative sequelae related to surgical avulsion of included mandibular third molars. A significant difference emerged in the pre- and post- versus post-administration of etoricoxib at the 2 h, 6 h, 24 h, 48 h, and 72 h follow-ups for the postoperative pain parameter. Similarly, a significant difference was found in the assessment of quality of life at the 3-day and 7-day follow-ups. However, no statistically significant results were observed between the two protocols of etoricoxib intake in the assessment of edema and trismus at the same time interval. The promising results of this preliminary analysis justify the need for future randomized controlled trials with a larger sample of patients.

## Data Availability

Research data supporting this publication are available from the corresponding author upon request.
